# The effect of fat mass on educational attainment: Examining the sensitivity to different identification strategies

**DOI:** 10.1016/j.ehb.2012.04.015

**Published:** 2012-12

**Authors:** Stephanie von Hinke Kessler Scholder, George Davey Smith, Debbie A. Lawlor, Carol Propper, Frank Windmeijer

**Affiliations:** a Department of Economics and Related Studies, University of York, York YO10 5DD, United Kingdom; b CMPO, University of Bristol, 2 Priory Road, Bristol BS8 1TX, United Kingdom; c MRC Centre for Causal Analysis in Translational Epidemiology (CAiTE), School of Social and Community Medicine, University of Bristol, Oakfield House, Oakfield Grove, Bristol BS8 2BN, United Kingdom; d Imperial College Business School, Imperial College London, South Kensington Campus, London SW7 2AZ, United Kingdom; e Department of Economics, University of Bristol, 8 Woodland Road, Bristol BS8 1TN, United Kingdom; f Centre for Microdata, Methods and Practice, Institute for Fiscal Studies, 7 Ridgmount Street, London WC1E 7AE, United Kingdom

**Keywords:** Instrumental variables, Fixed effects, ALSPAC

## Abstract

The literature that examines the relationship between child or adolescent Body Mass Index (BMI) and academic attainment generally finds mixed results. This may be due to the use of different data sets, conditioning variables, or methodologies: studies either use an individual fixed effects (FE) approach and/or an instrumental variable (IV) specification. Using one common dataset, the Avon Longitudinal Study of Parents and Children, and a common set of controls, this paper compares the different approaches (including using different types of IV's), discusses their appropriateness, and contrasts their findings. We show that, although the results differ depending on the approach, most estimates cannot be statistically distinguished from OLS, nor from each other. Examining the potential violations of key assumptions of the different approaches and comparing their point estimates, we conclude that fat mass is unlikely to be causally related to academic achievement in adolescence.

## Highlights

► We examine the effect of adolescent adiposity on educational outcomes. ► We compare the findings using different approaches used in the literature. ► We use an individual fixed effects as well as IV approach, with different sets of instruments. ► We conclude that adiposity is unlikely to causally affect academic achievement in adolescence.

## 1 Introduction

There is a growing interest in the relationship between individuals’ physical traits and their economic success. The literature has focused on three attributes: beauty (Hamermesh and Biddle, 1994), height (Case and Paxson, 2008, von Hinke Kessler Scholder et al., 2010) and body size (Cawley, 2004, Cawley and Spiess, 2008). The recent rise in body size across a large set of countries (OECD, 2007) makes the latter particularly pertinent. Studies estimating the effect of Body Mass Index (BMI) or obesity on economic outcomes such as wages generally find obesity to be associated with lower wages, at least for white females (Cawley, 2004).^1^ Studies that focus on children or adolescents and their academic achievement however, report conflicting findings. Some find that BMI is inversely associated with academic test scores, though possibly only for girls (Ding et al., 2006, Sabia, 2007, Averett and Stifel, 2010), whilst others report no evidence of association in either gender (Fletcher and Lehrer, 2008, Kaestner and Grossman, 2009).

It is possible that the differences between studies are overstated: there may be no true differences in associations across studies, but *p*-values for associations may vary due to different sample sizes. Differences between studies may also arise because of different associations in different populations, the use of different conditioning variables, or different methodologies. To deal with the possible endogeneity of BMI, the literature has generally used either an individual fixed effects (FE) and/or instrumental variable (IV) approach. In this paper, we use one common dataset and control for the same extensive set of covariates. We focus on the effect of children's adiposity (fat mass, as measured by a dual-energy X-ray absorptiometry (DXA) scan) on their academic performance. The use of a direct measure of fat mass is one of the strengths of this paper, addressing a recent call for the use of more accurate measures of obesity than the generally used BMI based on self-reported height and weight, or even BMI based on measurements (Burkhauser and Cawley, 2008, Burkhauser et al., 2009). We acknowledge, however, that there are very strong correlations between BMI and fat mass, with similar associations of both with cardiovascular risk factors, including in this dataset (Lawlor et al., 2010).

To account for the possible endogeneity of adiposity, we follow the existing literature and use an individual FE approach as well as IV. Within the latter, we distinguish between three different sets of instruments that have been used in this literature. We discuss their appropriateness, compare their performance and contrast their findings. First, we use the mother's pre-pregnancy BMI to instrument for the child's fat mass (as in Sabia, 2007, Averett and Stifel, 2010). Second, we use the child's fat mass in previous periods as instruments for the child's current fat mass (Kaestner and Grossman, 2009). Third, we specify two previously used genetic markers as instruments for current fat mass (von Hinke Kessler Scholder et al., 2011a). Although one may argue that (some of) these identification strategies are problematic, they have all been used in the literature and hence we compare each of them in this study.

OLS results show that more adipose children perform worse in school tests compared to their leaner counterparts. These findings are robust to an individual FE specification. We show that the IV results differ depending on the instrument set chosen. Accounting for the endogeneity of adiposity by using maternal pre-pregnancy BMI yields large negative estimates, suggesting that greater adiposity is associated with poorer school outcomes, possibly to a larger extent than that observed using OLS analysis. Using children's lagged fat mass to instrument for current fat mass leads to (patterns of) estimates that are very similar to the OLS findings, but with slightly larger standard errors. Accounting for the endogeneity of fat mass using genetic markers shows somewhat more ambiguous results, with point estimates that are sometimes smaller and sometimes larger than the OLS results. With the large standard errors however, we cannot reject the null of no effect.

The different approaches make different assumptions, which may or may not be valid in this context. OLS for example, is likely to be subject to residual confounding, and a FE approach does not deal with reverse causation, nor does it deal with time-varying unobservables that affect both fat mass and child outcomes. In addition, we show that the two non-genetic instrument sets are associated with several child and family background characteristics that are also associated with children's educational outcomes. This suggests that – in this context, where the main concern relates to unobserved confounding rather than reverse causation as we discuss below – they do not satisfy the exclusion restriction criteria required for a valid instrument. Hence, when examining the effects of children's fat mass/BMI on their outcomes, we call for a cautious use of these measures as instrumental variables for children's adiposity. In contrast, we show that the genetic variants are generally unrelated to the background characteristics that are associated with children's educational attainment. Taken together, this suggests that the use of carefully chosen genetic variants as instrumental variables is least likely to obtain biased causal effects.

Nevertheless, most estimates cannot be statistically distinguished from OLS, nor from each other. Taking account of the potential violations of key assumptions when addressing causality using the different approaches and comparing their point estimates, we conclude that fat mass is unlikely to be causally related to academic achievement in adolescence.

## 2 The previous literature

Sabia (2007) examines 14–17 year-olds from the National Longitudinal Study of Adolescent Health (Add Health) and finds a negative relationship between white girls’ BMI and their educational achievement. These results are robust to an individual FE approach and to a specification using IV, with mother and father's self-reported obesity status as instruments. But the estimates of any effect are small: it takes a weight difference of approximately 150 pounds (68 kg) for there to be a half-letter grade difference in Grade Point Average, all else equal. Averett and Stifel (2010) examine the effects of being overweight on educational attainment using data on the children of female cohort members of the National Longitudinal Survey of Youth 1979 (C-NLSY79), from 1986 to 2002. Focusing on elementary school-age children aged 6–13, they show that overweight children have lower educational outcomes compared to children of a healthy weight. This finding remains when using individual FE or IV, with mother's pre-pregnancy BMI and its square as instruments.

Kaestner and Grossman (2009) examine 5–12 year-olds of the C-NLSY79 between 1986 and 2004. They regress the change in educational attainment over two years on indicators representing the child's under- and overweight status and use IV, specifying the child's lagged BMI percentiles as instruments for current weight. They find no evidence that children's academic progress is affected by their weight.

The main aim in von Hinke Kessler Scholder et al. (2011a) is to provide a discussion of the conditions that need to be met for genetic markers to be used as instruments. Their application uses the Avon Longitudinal Study of Parents and Children (ALSPAC) to examine the effects of children's fat mass on their academic achievement. They use the genetic variants *FTO* and *MC4R* as instrumental variables for fat mass as measured by a DXA scan. Using this so-called ‘Mendelian randomization’ approach (Davey Smith and Ebrahim, 2003), they find no evidence of a causal effect of fat mass on educational outcomes.^2^

The above studies all account for a similar ‘standard’ set of covariates (such as gender, age, birth weight, household composition, household income, maternal employment, education and age at birth), though Sabia (2007) and von Hinke Kessler Scholder et al. (2011a) exploit the more extensive set of background characteristics available in their data. This includes covariates such as parental preferences and investment in children, maternal mental health, and maternal or child alcohol and cigarette intake. von Hinke Kessler Scholder et al. (2011a) show however, that the addition of these variables does not lead to large changes in the point estimates. Hence, though possible, we do not expect the differences in covariates to explain all differences in findings between these studies.

There are many other studies that examine the relationship between body weight and educational outcomes or IQ. Some examine the effects of education/IQ on body weight, whilst others explore the effect of body weight on education/IQ (see e.g. Sorensen et al., 1983, Li, 1995). Only few studies are prospective (see e.g. Lawlor et al. (2006) and references therein), but with most using a cross-sectional design, the direction of the estimated associations is unclear. Although a FE specification does not deal with this reverse causation, the IV approach may be able to depending on when the instrument, BMI and outcome of interest are measured.

## 3 Data

Our data are from a cohort of children born in one geographic area (Avon) of England. Women eligible for enrolment in the population-based ALSPAC study had an expected delivery date between 1 April 1991 and 31 December 1992. Approximately 85% enrolled, leading to about 14,000 pregnancies. The Avon area is broadly representative of the UK, though individuals are slightly more affluent than the general population in that they are less likely to live in rented accommodation and to have a father in a manual occupation. The Avon area does not show any marked differences in factors such as single parenthood, parental education, mobility, parental smoking, birth weight, mental and physical disabilities, and the proportion living in rural areas compared to the whole of Great Britain (Golding et al., 2001).^3^ The variables in ALSPAC include a wide range of child and family background characteristics, including information on child health, child development and family inputs. Data are collected from various sources, such as in-depth interviews, self-completed questionnaires, biological samples, and linkage to medical and school records. This allows us to examine the very detailed information on child and family background.

We observe 12,620 children who returned at least one questionnaire. Of these, 642 were excluded because either their mother or father is of non-white ethnic origin, leaving 11,978 potential participants.^4^ Our sample selection process is as follows. First, we select those children for whom we observe their adiposity as measured by a DXA scan. This was recorded at specially designed clinics, which all children were invited to attend. As not all children attended these clinics, our sample sizes drop to 6078. Second, as we wish to compare the different sets of instruments used in this literature, we drop those observations with missing information on any of the three instrument sets, leaving us with 3728 children. Finally, we restrict the sample to those children for whom we observe their educational outcomes, leading to a final sample size of 3001. We deal with missing values on other covariates using multivariate imputation. Table 1 compares the original sample to our estimation sample; we discuss this in more detail after discussing the variables used in the analyses.

**Table 1:** Descriptive statistics for the full sample and the estimation sample.

Variable	(1) Full sample		(2) Estimation sample		(3) *p*-Value, two-sample mean test
	Mean	Std. dev	Mean	Std. dev	
*Outcome variable*					
KS3 score	100.00	10.00	103.30	8.49	<0.001
KS2 score	100.00	10.00	102.40	8.39	<0.001
Variables of interest					
Fat mass, age 11	100.00	10.00	99.70	9.80	0.170
Fat mass, age 9	100.00	10.00	99.70	9.69	0.164

*Contextual variables*					
Percent female	0.48	0.50	0.51	0.50	0.003
Birth weight	3395	560	3416	540	0.053
Older siblings (0, 1, 2 or more)	0.75	0.76	0.71	0.73	0.008
Younger siblings (0, 1, 2 or more)	0.46	0.63	0.53	0.65	<0.001
Ln (income)	5.28	0.49	5.35	0.44	<0.001
Mother's education	2.26	0.92	2.41	0.88	<0.001
Grandmother's education	1.71	0.78	1.75	0.76	0.014
Grandfather's education	1.81	0.81	1.86	0.77	0.003
Raised by natural father	0.92	0.27	0.95	0.22	<0.001
Father's social class	3.06	1.31	3.02	1.29	0.134
Mother's age	3.21	0.99	3.41	0.89	<0.001
Mother works PT, at 21 months	0.36	0.48	0.41	0.49	<0.001
Mother works FT, at 21 months	0.10	0.30	0.11	0.31	0.119
Partner is employed, 21 months	0.97	0.44	0.95	0.32	0.004
IMD	21.26	15.25	18.98	13.62	<0.001

*Parental health and behaviour*					
Mother smoked during pregnancy	0.24	0.43	0.17	0.37	<0.001
Mother drank alcohol during pregnancy	0.55	0.50	0.56	0.50	0.327
Intensity of breastfeeding	1.76	1.24	1.92	1.19	<0.001
Mother's ‘locus of control’	100.11	9.62	98.45	9.39	<0.001
Mother's CCEI	13.65	7.74	12.67	7.14	<0.001
Mother's EPDS	6.95	4.83	6.24	4.50	<0.001
Mother's teaching score	7.01	1.09	7.04	0.90	0.121
Interest in child's development	0.69	0.21	0.69	0.20	1.000
Parent's activity score	27.73	4.95	27.90	4.53	0.087

*Instrumental variables*					
Mother's BMI	100.00	10.00	100.16	9.63	0.425
Child weight categories	3.00	0.77	2.99	0.76	0.552
*FTO* (categorical, with values 0, 1 and 2)	0.79	0.69	0.79	0.69	0.788
*MC4R* (binary, values 0, 1)	0.43	0.49	0.42	0.49	0.340
Number of observations	12.620			3001	

*Note*: The descriptive statistics of the full sample are based on a maximum of 12.620 observations if the variable reported in the column has no missing values on any observations.

### 3.1 Measures of academic achievement

Our main outcome measure is the child's exam result on the Key Stage 3 (KS3) test. The KS3 exam is a nationally set exam, taken by all 14-year-olds in English state (public) schools.^5^ Children's scores for three subjects (English, maths and science) are obtained from the National Pupil Database, a census of all pupils in England within the state school system, which is matched with ALSPAC records. We use an average score for the three subjects, standardised on the full sample of children for whom data are available, with mean 100 and standard deviation 10. When we use two measures of academic achievement in the FE analysis, we also include the child's Key Stage 2 (KS2) result, a similar nationally set exam taken at age 11.

### 3.2 Child fat mass

We measure child adiposity by the child's body fat mass (adjusted for gender, age in months, height and height squared), as determined by a dual-energy X-ray absorptiometry (DXA) scan. This method scans the whole body, dividing it into body fat, lean tissue mass, and bone density. Our focus is on the child's fat mass at age 11. We standardise this on the full sample of children for whom data are available, with mean 100 and standard deviation 10.

### 3.3 The instrumental variables

We compare three sets of instruments. First, we use the mother's pre-pregnancy BMI and its square. To make the maternal BMI distribution comparable with the child's fat mass distribution, we standardise it to have mean 100, standard deviation 10. Second, we use percentiles of the child's fat mass in a previous period. More specifically, as in Kaestner and Grossman (2009), we use the percentiles 0–5, 6–15, 16–84, 85–94 and 95–100 of the child's adiposity distribution at age 9 as instruments for fat mass at age 11. Third, we use two genetic markers that have been consistently shown to relate to (child) BMI and fat mass: *FTO* (rs9939609) and *MC4R* (rs17782313).^6^ The genetic model for *FTO* is additive, meaning that each risk allele (A) affects the phenotype by a similar amount.^7^ Hence, we enter this as one variable with three categories: no risk alleles (homozygous TT), one risk allele (heterozygous AT) and two risk alleles (homozygous AA). The genetic model for *MC4R* is dominant, meaning that the presence of any risk allele – either one or two – is associated with a similar increase in adiposity (Timpson et al., 2009a). We therefore specify this as a binary variable indicating whether the child carries at least one risk allele (C); i.e. we compare individuals with genotype CC or CT to those with genotype TT.

### 3.4 Control variables

We observe an unusually rich set of child and family background characteristics that we include as covariates as they may be related to both adiposity and the child's educational performance. In addition, we use these to test whether they differ for the different instrument sets used.^8^ As the main aim of this paper is to compare the different approaches and contrast their findings, we use the same set of covariates in all specifications and report the unadjusted as well as adjusted OLS and IV estimates.^9^

We control for the child's birth weight and for the number of older and younger siblings under 18 in the household. As children's educational outcomes are known to differ with within-year age, the analyses include binary indicators for children's age (in months). We include several controls for socio-economic position: we account for the log of family income and its square, four binary indicators for mother's educational level, the mother's parents’ educational level, an indicator for whether the child is raised by the natural father, binary indicators for the family's social class, maternal age at birth, and parental employment status when the child is 21 months. We also include a measure of small (local) area deprivation: the Index of Multiple Deprivation (IMD).^10^

In addition to these generally observed controls, our data allow us to also account for further measures of mother's health and behaviour, which may be correlated to both children's adiposity and educational attainment. We include two binary variables that measure whether the mother smoked or drank alcohol in the first three months of pregnancy and account for ordered indicators for the intensity of mother's breastfeeding (never, <1month, 1–3 months and 3+ months). We include the mother's ‘locus of control’, a psychological concept that describes whether individuals attribute successes and failures to internal or external causes. Those with an internal (low) locus of control see themselves as responsible for the outcomes of their actions. Those with an external (high) locus of control believe that successes and failures are chance-determined. We include two measures of maternal mental health to account for possible confounding and control for several measures of parental involvement or interest in the child's development.^11^

Finally, we control for school fixed effects to account for possible clustering by schools of children's outcomes and calorie intake and expenditure patterns (such as via school meals and physical activity). We do not include these in all specifications, as this drops an additional 155 children from our sample. For these children, we observe no other pupils in their school; hence, we cannot estimate a school effect.

Column 1 in Table 1 presents summary statistics for the full sample of children for whom data is available; column 2 shows the statistics for the estimation sample. The former is based on a maximum of 12,620 children if the variable has no missing values on any observations; our estimation sample consists of 3001 children. There is statistical evidence of differences between the two samples for many of the characteristics, but for most the actual magnitude of difference is small. For example, the average Key Stage scores in the final sample are higher than that in the original sample, there are more girls, and babies have a higher average birth weight. The estimation sample is also of higher socio-economic position: they have higher incomes, mothers and grandparents are better educated, and live in less deprived areas. Mothers in the estimation sample are also less likely to have smoked during pregnancy, and have breastfed for longer. Finally, they have a lower locus of control and are in better mental health. Note however, that there is no strong statistical evidence that the means of all three sets of instrumental variables differ between the samples, suggesting that the attrition is unrelated to the different instrument sets.^12^

## 4 Estimation strategy and hypotheses

We examine the impact of children's adiposity on their educational outcomes. We begin with a simple linear model:

(1) $$S_{i,14}=\beta_{0}+\beta_{1}A_{i,11}+u_{i,14}\text{,}$$

where child *i*'s exam result at age 14 ($S_{i,14}$, the KS3 exam) is a function of the child's adiposity measured at age 11 ($A_{i,11}$). The term $u_{i,14}$ represents the unobserved component, which includes both unobserved child attributes and unobserved parental/family behaviour. The parameter of interest *β*_1_ measures the average relationship between child adiposity and academic achievement. We augment Eq. (1) to account for the set of child and family background characteristics and indicators for parental health and behaviour described above, which allow us to explore how the relationship between child adiposity and academic achievement changes when controlling for various observed inputs in the child education production function.^13^

The possible endogeneity of child adiposity is characterised by the fact that the unobservable confounders $u_{i,14}$ determine educational outcomes $S_{i,14}$, but also determine $A_{i,11}$, leading to biased OLS estimates. The bias is likely to be negative if we assume that excess fat mass is negatively related to children's educational outcomes.

The existing literature generally attempts to deal with the endogeneity problem by either estimating child FE models when children are observed multiple times, or by using IV. The fixed effects specification deals with the endogeneity problem only if the unobserved factors that jointly affect child adiposity and educational outcomes are constant over time. Any time-varying unobservables such as changes in children's peer groups, and changes in family or household circumstances that affect both school performance and adiposity (gain) may therefore still bias the estimates. Additionally, the fixed effects model does not deal with any reverse causality running from school outcomes to fat mass. When we use FE below, the outcome of interest includes two exam results: KS2 and KS3, taken at age 11 and 14 respectively. Adiposity is measured at ages 9 and 11.

The IV method estimates the average causal effect $\beta_{1}$ in (1) by introducing instrumental variables *Z*_*i*_ that are associated with $A_{i,11}$, but only associated with $S_{i,14}$ indirectly through its association with $A_{i,11}$. In the absence of a constant treatment effect, Angrist et al. (2000) specify the assumptions needed for the standard linear IV estimator in (1) to identify the average causal response within a potential outcomes framework. These are: A1 – independence and exclusion, A2 – a non-zero effect of the instrument on adiposity, and A3 – monotonicity.^14^

We focus on the first two. Assumption A1, independence and exclusion, implies that the instrument is as good as randomly assigned, and that the potential outcomes are unchanged by the presence or absence of the instrument. Assumption A2, the non-zero effect of the instrument on adiposity, refers to the (first stage) regression of A_*i*,11_ on *Z*_*i*_ to be non-zero, sometimes referred to as the relevance assumption.

We compare three instrument sets that have previously been used in this literature: the child's adiposity in previous periods, maternal BMI in a previous period, and the child's genetic variants.^15^ Within the IV approach, the assumptions are identical for the three instrument sets. Although they are different from the fixed effects specification, which in turn could result in different estimates, they both aim to estimate a causal effect of adiposity on educational attainment. Hence, large differences between the different approaches would suggest it is important to identify possible drivers of these differences. Any differences may be due to the identification of different Local Average Treatment Effects (LATE's), but may also be caused by a violation of one or more of the above assumptions.

### 4.1 The child's adiposity in previous periods

The use of the child's adiposity at earlier ages is often justified by arguing that this deals with problems of reverse causation, as current outcomes cannot affect previous adiposity. However, current educational attainment is related to earlier attainment, which may have affected earlier adiposity. Hence, we cannot completely rule out any reverse causality.^16^

Relating the instrument to the IV assumptions, the child's lagged adiposity is likely to be related to the child's current adiposity, satisfying the relevance assumption. However, whether it satisfies the independence and exclusion assumption is debatable. Depending on the lag used, the correlation between children's past and current adiposity, can be as high as 0.95.^17^ Such substantial correlation suggests that the child's earlier fat mass is more or less a perfect predictor of its current fat mass and raises doubts about its use as an IV. For previous fat mass to be a valid instrument, the component of fat mass that is uncorrelated to lagged fat mass needs to contain all of the correlation with the unobserved characteristics of school performance *u*_*i*_,_14_. Put another way: all factors contributing to the high correlation between fat mass and lagged fat mass must be unrelated with these unobserved components. With the (unobserved) family environment being an important determinant of both fat mass and educational outcomes, there are various situations that can violate this assumption. For example, high unobserved time discount rates can decrease children's school performance and increase their fat mass. If this affects fat mass at all ages, both lagged and contemporaneous fat mass will be endogenous. With the child's lagged fat mass being more or less a perfect predictor of current fat mass, our prior is for the IV estimates that use the child's lagged fat mass as the instruments to be very similar to the OLS estimates.

Note however that, if fat mass is actually exogenous, the use of lagged fat mass as instrumental variables will also lead to results that are very similar to OLS. Hence, similarity of the IV and OLS findings does not *in itself* imply that fat mass in endogenous. In other words, similarity of the two models is not *necessarily* a sign of a poor instrument.

### 4.2 Maternal pre-pregnancy BMI

The evidence of a genetic component in weight is often used to justify the choice of maternal or paternal BMI as a ‘quasi-genetic’ instrument (Sabia, 2007, Averett and Stifel, 2010). Although its use as an instrument likely satisfies the relevance assumption, whether it satisfies the exclusion restriction is again debatable. Maternal (lagged) BMI is likely to be correlated with family resources, unmeasured preferences or choices, and educational inputs (Kaestner and Grossman, 2009). For example, discrimination against obese females in the labour market (Cawley, 2004) can affect the family's financial resources that are available for inputs into the child education production function.

Hence, as in all IV analyses, the validity of the instrumental variables will depend on the context and research question. If the main concern relates to reverse causation, for example when studying the effects of BMI on a chronic disease, maternal BMI may be a valid instrument. In contrast, if the main concern relates to unobserved confounding (as is the case here), maternal BMI may have a direct effect on the outcome of interest, invalidating its use as an IV.

If the relationship between children's fat mass and educational outcomes is driven by, for example, unobserved family environments or socio-economic position, the covariance between mother's BMI (*M*_*i*_) and the unobservables, i.e. the numerator of the IV bias term $\text{Cov}(M_{i}u_{i,14})/\text{Cov}(M_{i}A_{i,11})$, is likely to have the same sign as the covariance between the child's own adiposity and the unobservables. In fact, as a longer exposure to the (unobserved) environment for the mother may lead to stronger correlations with BMI compared to that for the child, the former may actually be larger than the latter. We examine this indirectly in the descriptive statistics presented below. In addition, this would suggest that the IV estimates that use mother's BMI as the instruments will be more negative compared to the OLS estimates and compared to the findings that use the child's lagged adiposity as the instruments. We investigate this below.

### 4.3 The child's genetic markers

The specific choice of genetic markers as instrumental variables is crucial in Mendelian randomization experiments. As in all IV studies, an incorrect choice may lead to violations of the IV assumptions and bias the estimates. The two genetic variants used here have been consistently shown to relate to BMI and fat mass: *FTO* and *MC4R* (see footnote 6). We use the standard statistical tools to examine the strength of our instruments below, i.e. to test whether the relevance assumption holds.

As with the other instruments discussed above, we cannot directly test whether the exclusion restriction holds. However, we examine this indirectly. The assumption could be violated if, for example, the mechanism through which the genetic variant affects fat mass involves changes in behaviour or preferences that also directly affect the outcome. The current evidence suggests that the variants are associated with an increased consumption of fat and energy (see e.g. Timpson et al., 2008). The literature suggests that the variants increase food intake due to diminished satiety (Wardle et al., 2008), rather than through pathways that directly affect the outcome of interest. This has been confirmed in mice studies, showing that the increased body mass primarily results from increased food intake (Church et al., 2010).

To examine any potential alternative effects of the instruments, one can search databases such as PubMed to explore whether the instruments have been associated with any other (health) conditions or characteristics that could violate the IV assumptions. For example, one study finds that *FTO* increases mortality independent of fat mass (Zimmerman et al., 2009), whilst another shows a relationship with prostate cancer (Lewis et al., 2010). If valid, these could question the validity of our IV strategy. In this case however, we know that the sample size of the study by Zimmerman is small, and there are much larger studies showing the expected effects of *FTO* on (e.g.) coronary heart disease (Nordestgaard et al., 2010) through their effects on adiposity (rather than *FTO* having a direct effect on the outcome). In addition, the magnitude of the effect of *FTO* on prostate cancer is expected, given its effects on BMI and the effects of BMI on prostate cancer. More generally, *FTO* shows consistent effects on various obesity-related risk markers, including hypertension (Timpson et al., 2009a), bone mass (Timpson et al., 2009b), and diabetes-related metabolic traits (Freathy et al., 2008), suggesting that the variant affects the outcome of interest through its effect on fat mass/adiposity; i.e. *FTO* is acting as an instrument for adiposity rather than it violating the IV assumptions.

Another way in which the exclusion restriction could be violated is if the genetic variant has multiple functions (i.e. if it is pleiotropic), which directly affect the outcome of interest. Likewise, if the variant is co-inherited with another genetic variant (i.e. if it is in linkage disequilibrium (LD)), violation of this assumption depends on the effect of the co-inherited variant.^18^ Although the specific biological pathway between our instruments and fat mass is unknown, there is substantial evidence that they are related to a diminished satiety and increased food intake.^19^ As the exact biological, or molecular, mechanism is unknown however, we cannot with complete certainty claim that the instruments are not complicated in any other mechanism that may affect the outcome of interest. If they are, it could invalidate the IV approach and lead to biased estimates. In other words, as with any (genetic or non-genetic) instruments, the validity of the exclusion restriction will never be known with complete certainty and can only be examined indirectly. We do this by correlating the genetic variants with a wide range of maternal, family and child characteristics and behaviours, comparing the performance of the genetic variants to the other instrument sets discussed above.^20^

As *FTO* and *MC4R* are associated with an increased consumption of fat and energy, this suggests that they may also explain weight *gain* between two time periods. If so, it would suggest that one may be able to use the genetic variants as instruments for weight gain in a fixed effects IV regression. The genetic instruments indeed explain some variation in weight gain. However, 11 year-olds who are homozygous for the rare allele of *FTO* are on average two kilograms heavier than those homozygous for the common allele. For adults, the difference between the two groups is about 2.5 kg. Hence, the correlation between the genetic variants and the *gain* in weight is not sufficient for an IV analysis, especially when the individuals are observed only two years apart; we therefore do not pursue this further.

## 5 Results

### 5.1 Descriptive statistics

Table 2 shows the bivariate correlations between each of the covariates and the different instrumental variables. This shows the extent to which the three instrument sets are (unconditionally) associated with the various (observed) child and family background characteristics used in the analyses. Although we would expect some significant differences by chance, we should not observe any strong patterns between the observables and the instruments. However, the table shows several significant relationships and patterns in the data: BMI in mothers and adiposity in children are both inversely associated with income, mother's education, grandmother's education, grandfather's education and social class. Similarly, they are positively associated with deprivation (IMD), mother's locus of control and mother's mental health problems (CCEI). This suggests there is a strong socio-economic gradient for both non-genetic instrument sets. In addition, the table shows that the majority of these covariates have stronger associations with mother's BMI than with the child's fat mass, confirming our hypothesis that the IV estimates that use mother's BMI as the instruments are likely to be more negative compared to both the OLS estimates and to the findings that use the child's lagged adiposity as the instruments.

**Table 2:** Coefficients (std. err) of the bivariate correlations, obtained from separate regression of the indicators presented in the first column on each instrumental variable.

	Mother's BMI	Child's fat mass, age 9	*FTO*	*MC4R*
Girl^a^	−0.001	−0.000	0.010	−0.034*
	(0.001)	(0.001)	(0.013)	(0.018)
Birth weight	7.718***	1.905*	11.596	−41.152**
	(1.227)	(1.057)	(14.280)	(20.038)
Older siblings (0, 1, 2 or more)	0.004***	0.001	−0.015	0.005
	(0.001)	(0.001)	(0.019)	(0.027)
Younger siblings (0, 1, 2 or more)	−0.003**	−0.002	0.030*	0.025
	(0.001)	(0.001)	(0.017)	(0.024)
Ln (income)	−0.003***	−0.003***	0.010	−0.006
	(0.001)	(0.001)	(0.012)	(0.016)
Mother's education	−0.011***	−0.010***	0.023	0.012
	(0.002)	(0.002)	(0.023)	(0.033)
Grandmother's education	−0.008***	−0.006***	−0.001	0.032
	(0.001)	(0.001)	(0.020)	(0.028)
Grandfather's education	−0.009***	−0.007***	0.072***	0.023
	(0.001)	(0.001)	(0.020)	(0.029)
Raised by natural father^a^	0.001**	−0.001*	−0.002	0.018**
	(0.000)	(0.000)	(0.006)	(0.008)
Father's social class	0.015***	0.007***	−0.084**	−0.056
	(0.002)	(0.002)	(0.033)	(0.047)
Mother's age	−0.002	−0.003**	−0.007	−0.024
	(0.002)	(0.002)	(0.024)	(0.033)
Mother works PT, at 21 months^a^	0.001	−0.001	−0.008	0.018
	(0.001)	(0.001)	(0.013)	(0.018)
Mother works FT, at 21 months^a^	0.001	−0.000	0.003	−0.030***
	(0.001)	(0.001)	(0.008)	(0.011)
Partner is employed, 21 months^a^	−0.000	0.000	−0.009	0.013
	(0.001)	(0.001)	(0.007)	(0.010)
IMD	0.107***	0.145***	0.376	−0.752
	(0.026)	(0.027)	(0.359)	(0.499)
Mother smoked during pregnancy^a^	0.000	0.003***	−0.008	0.002
	(0.001)	(0.001)	(0.010)	(0.014)
Mother drank alcohol during pregnancy^a^	0.000	−0.001	0.002	−0.023
	(0.001)	(0.001)	(0.013)	(0.018)
Intensity of breastfeeding	−0.016***	−0.015***	0.047	0.119***
	(0.002)	(0.002)	(0.032)	(0.044)
Mother's ‘locus of control’	0.095***	0.102***	0.364	0.319
	(0.018)	(0.018)	(0.250)	(0.345)
Mother's CCEI	0.028**	0.027*	0.136	0.473*
	(0.014)	(0.014)	(0.187)	(0.262)
Mother's EPDS	−0.005	0.007	0.070	0.185
	(0.009)	(0.009)	(0.119)	(0.166)
Mother's teaching score	−0.002	−0.001	0.004	−0.068**
	(0.002)	(0.002)	(0.024)	(0.034)
Interest in child's development	−0.000	−0.000	0.005	0.005
	(0.000)	(0.000)	(0.005)	(0.007)
Parent's activity score	−0.017**	−0.004	−0.042	0.213
	(0.009)	(0.009)	(0.115)	(0.168)
Number of observations	3001	3001	3001	3001

a Binary indicators.

* *p* < 0.10.

** *p* < 0.05.

*** *p* < 0.01.

Columns 3 and 4 show that, for example, *FTO* is positively related to grandfather's education and social class, and that those who carry one or two *MC4R* risk alleles are more likely to be raised by their natural father, their mothers are less likely to work full-time, and are more likely to breastfeed longer. However, there are no clear patterns such as those observed for mother's BMI and children's fat mass, suggesting that the genetic variants are largely unrelated to these background characteristics.^21^

Although we observe this very rich set of covariates and can control for it in the specifications described above, there may still be a host of other family or child factors that are related to both obesity and outcomes, but are unobserved to the researcher. With Table 2 showing strong socio-economic gradients for maternal BMI and the child's fat mass, there is no compelling reason to believe that there is no such gradient between these instruments and other unobservables.

### 5.2 OLS

**Fig. 1:**
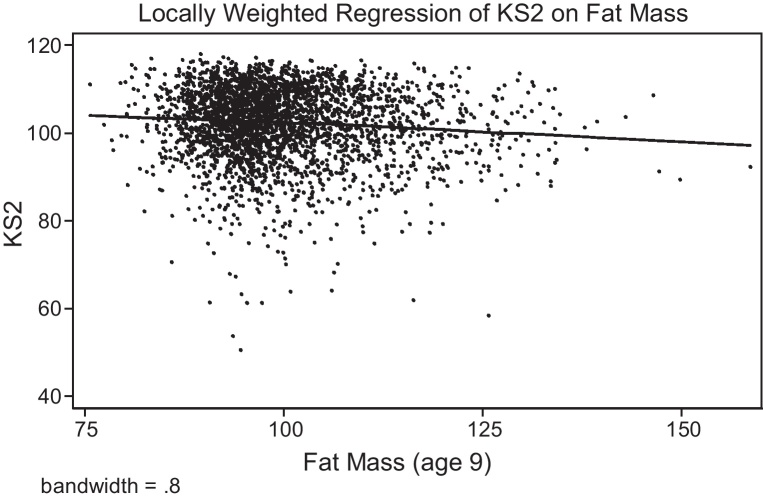
Non-parametric regression of KS2 on adiposity at age 9.

**Fig. 2:**
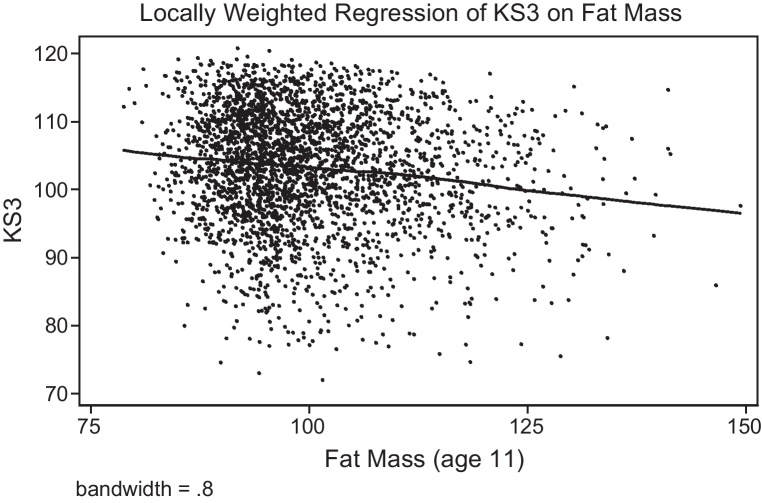
Non-parametric regression of KS3 on adiposity at age 11.

We begin by examining the non-parametric relationship between the child's fat mass at age 11 and educational attainment at age 14 (KS3 scores), and between fat mass at age 9 and educational attainment at age 11 (KS2 scores). Fig. 1, Fig. 2 show a clear negative relationship for both, which is linear over the full range of the adiposity distribution. Hence, we start with an OLS regression of KS3 (age 14) on the child's fat mass (age 11); this is presented in columns 1–5 of Panel A in Table 3, with each column subsequently adding more control variables. The raw correlation between educational attainment and fat mass is negative, with a one standard deviation increase in fat mass associated with a 0.11 standard deviation decrease in test scores (column 1). We augment Eq. (1) to account for the contextual variables (column 2) and mother's health and behaviour (column 3). This brings the estimate closer to zero, but it remains negative and statistically significant. Column (4) is similar to (3), but uses the slightly smaller sample size that corresponds with the sample when including school fixed effects, which is presented in column 5. Panel B presents the results for the regressions of KS2 (age 11) on fat mass at age 9, showing slightly smaller estimates, though with a similar pattern. In summary, all OLS specifications suggest that there is a negative correlation, albeit a small one, between the child's fat mass and educational attainment.

**Table 3:** OLS and FE of academic achievement (KS scores) on child adiposity (fat mass).

	OLS					Child FE
	(1)	(2)	(3)	(4)	(5)	(6)
**A. KS3 (at 14)**						
Fat mass (at age 11)	−0.110***	−0.051***	−0.042***	−0.043***	−0.037**	−0.030*
	(0.016)	(0.014)	(0.014)	(0.014)	(0.015)	(0.017)
*R*-squared	0.02	0.26	0.29	0.28	0.32	0.06
Number of children	3001	3001	3001	2846	2846	3001
*N* × *T*						6002

**B. KS2 (at 11)**						
Fat Mass (at age 9)	−0.074***	−0.030**	−0.025*	−0.027*	−0.025*	−0.030*
	(0.015)	(0.014)	(0.014)	(0.015)	(0.015)	(0.017)
*R*-squared	0.01	0.21	0.23	0.23	0.26	0.06
Number of children	3001	3001	3001	2846	2846	3001
*N* × *T*						6002

Contextual variables	−	Yes	Yes	Yes	Yes	Yes^a^
Mother's health and behaviour	−	−	Yes	Yes	Yes	Yes^a^
School fixed effects	−	−	−	−	Yes	−

*Notes*: Robust standard errors in parentheses. The contextual variables include: birth weight, number of older and younger siblings under 18, age in months, log equivalised family income and its square, mother's educational level, mother's parents’ educational level, raised by natural father, social class, maternal age at birth, parents’ employment status, and IMD. Mother's health and behaviour include: mother's smoking and drinking during pregnancy, breastfeeding, mother's ‘locus of control’, two measures of maternal mental health (EPDS and CCEI), parental involvement or interest in the child's development, and parents’ engagement in active (outdoor) activities with their child. Including school fixed effects drops 155 children from the analysis, as we only observe a single child in their school.

a The child fixed effects analysis drops all time-invariant indicators, hence we only include the child's age as a covariate.

* *p* < 0.10.

** *p* < 0.05.

*** *p* < 0.01.

### 5.3 Fixed effects

We now turn to the individual fixed effects analysis. We use the child's exam result on the KS2 and KS3 tests as the dependent variables, and specify the child's fat mass at ages 9 and 11 as the variables of interest. Hence, we perform a fixed effects analysis of the Key Stage test score on the lagged measure of child adiposity. As most of our covariates are either time-invariant (such as birth weight or maternal education) or not observed multiple times (such as family income), these drop out of the analysis. Hence, our only control variable in the FE analysis is the child's age in months at the time the Key Stage tests are taken. The results are presented in column 6 of Table 3 (panels A and B are identical). The child FE estimate is similar to the estimate obtained from OLS, again suggesting that fat mass negatively affects children's educational outcomes. However, as discussed above, the fixed effects analysis does not account for time-varying unobservables that affect both child fat mass and educational performance. Our next step is therefore to examine the IV strategy.

### 5.4 Instrumental variables

We explore three sets of instruments. First, following Averett and Stifel (2010) and Sabia (2007), we instrument child fat mass with maternal pre-pregnancy BMI and BMI squared. Second, as Kaestner and Grossman (2009), we use the child's lagged under- and overweight categories (the percentiles 0–5, 6–15, 16–84, 85–94 and 95–100 of the age 9 fat mass distribution) as instruments for later fat mass.^22^ Finally, we present the findings using the child's genetic variants *FTO* and *MC4R* as instruments for fat mass.

Table 4 presents the specification tests for the first-stage regressions using the three sets of instruments. Whether or not we control for the background characteristics, all instruments are strongly associated with child fat mass, as shown by the *F*-statistic of IV strength. This is particularly large when using the child's lagged fat mass (*F* = 1496), and maternal BMI (*F* = 127), but remains considerable when using the genetic variants (*F* = 19). The LM test for under-identification has large values in all specifications, indicating that the models are identified.

**Table 4:** First stage specification tests of the instrumental variable regressions of child adiposity (fat mass).

	(1) Fat mass, age 11	(2) Fat mass, age 11	(3) Fat mass, age 11
**A. maternal BMI as IV**			
IV strength, *F*-statistic	145.0	127.4	127.1
Under identification LM test^a^	229.8	210.0	209.7

**B. child's lagged fat mass as IV**			
IV strength, *F*-statistic	1661.7	1513.8	1496.0
Under identification LM test^a^	746.7	740.0	744.7

**C. genetic markers as IV**			
IV strength, F-statistic	16.0	20.2	19.1
Under identification LM test a	31.4	39.5	37.4

Contextual variables	–	Yes	Yes
Mother's health and behaviour	–	–	Yes

*Notes*: All controls included. Number of observations in all specifications is 3001.

a The Kleibergen-Paap LM statistic for under-identification.

Table 5 presents the second stage regression results; panels A–C refer to the different instrument sets. For comparison, column 1 replicates the OLS results without school fixed effects from Table 2, whilst columns 2–4 show the findings after instrumenting for fat mass. Controlling for all covariates, the OLS results show that fat mass negatively affects school performance. Using mother's pre-pregnancy BMI and BMI squared as instruments (panel A), this relationship remains negative and strong. The estimates suggest that one standard deviation increase in fat mass relates to a 0.12 standard deviation decrease in KS3 (column 4). In fact, the IV estimate is almost three times larger than the estimate from the OLS regression, suggesting that OLS underestimates the true effect. A Hausman test for the endogeneity of fat mass indeed rejects the null that fat mass is exogenous, suggesting that – if we believe that the IV assumptions are satisfied – we should rely on the IV estimates.

**Table 5:** Second stage IV results: KS3 on child adiposity (fat mass).

	OLS	2SLS		
	(1) KS3	(2) KS3	(3) KS3	(4) KS3
**A. maternal BMI as IV**				
Fat mass, age 11	−0.042***	−0.298***	−0.119***	−0.119***
	(0.014)	(0.051)	(0.046)	(0.046)
*p*-Value, Hansen J test		0.381	0.449	0.351
*p*-Value, Hausman test		0.000	0.115	0.074

**B. child's lagged fat mass as IV**				
Fat mass, age 11	−0.042***	−0.100***	−0.035**	−0.031*
	(0.014)	(0.019)	(0.018)	(0.017)
*p*-Value, Hansen J test		0.912	0.864	0.944
*p*-Value, Hausman test		0.377	0.165	0.318

**C. Genetic markers as IV**				
Fat mass, age 11	−0.042***	0.106	−0.098	−0.039
	(0.014)	(0.155)	(0.120)	(0.122)
*p*-Value, Hansen J test		0.528	0.418	0.644
*p*-Value, Hausman test		0.146	0.694	0.982

Contextual variables	Yes	–	Yes	Yes
Mother's health and behaviour	Yes	–	–	Yes
School fixed effects	–	–	–	–

*Notes*: Robust standard errors in parentheses. Number of observations is 3001.

* *p* < 0.10.

** *p* < 0.05.

*** *p* < 0.01.

Panel B, which uses the child's lagged adiposity as instruments for current adiposity, also shows negative effects of fat mass on KS3. Including the controls brings the estimate closer to zero, though it is still very similar to OLS. Finally, when genetic markers are used as instruments in panel C, we find somewhat ambiguous results, with point estimates that are sometimes smaller and sometimes larger than those in Panel B. As there are no large differences in the first stage regressions that do or do not control for covariates, the variability in the IV point estimates is likely to be due to the relative weakness of the genetic variants.^23^ Although the final IV estimate is very similar to that obtained by OLS, the large standard errors preclude us from rejecting the null of no effect. In fact, a Hausman test for the endogeneity of fat mass would suggest that – statistically speaking – we should rely on the OLS estimates rather than the IV (*p* = 0.892). However, we argue that any such conclusions should not be based on one test alone, but should take into account all available evidence discussed above, including the potential for violations of the model assumptions. With a very wide IV confidence interval, the comparison test between OLS and IV is unlikely to be very strong. However, even if one were willing to assume that including the wide range of variables in the OLS regression solves the endogeneity problem, the magnitude of the estimate shows that, if any, the effect is very small. The OLS confidence interval is [−0.07, −0.014], indicating that a one standard deviation increase in adiposity leads to a maximum of 0.07 standard deviations decrease in educational outcomes. This corresponds to a decrease in children's KS3 score from the median to the 47th percentile. Hence, even if there truly were an adverse effect of adiposity on educational outcomes, the magnitude of this effect is small.^24^

Despite the sometimes large differences in the point estimates of the different model specifications, the overidentification (Hansen J) tests in Table 5 do not suggest any of the instrument sets is actually invalid. This may be surprising given our priors discussed in Section 4 and given some of the large differences in point estimates. However, this may simply reflect the low power of the test.

The findings in panel A confirm Averett and Stifel (2010) and Sabia (2007), who use similar instruments. However, they are in contrast with the results in panel B and C. The only driver behind these differences is the choice of instruments, since the model specification and assumptions are identical in all other aspects. We cannot conclude however, that all differences in the existing literature are *necessarily solely* due to the use of different instrumental variables, as the studies control for different covariates, which can lead to different results. Nevertheless, the size and patterns of our estimates in panels A and B correspond to our priors as discussed in section 4: larger negative coefficients when using maternal BMI as the instruments (panel A), and similar coefficients to those found in OLS when using the child's lagged fat mass as the instruments (panel B).

Statistically speaking however, we cannot distinguish most estimates from the OLS estimates or from each other. This would suggest that we should rely on OLS rather than IV. Indeed, the use of maternal BMI as instrumental variables is likely to enhance any bias, the use of the child's lagged fat mass will be similar to OLS, and the use of genetic variants leads to very imprecise estimates. However, as discussed above, we are cautious about this interpretation, as the power of some of our analyses is low, particularly for the analyses that use the genetic variants as instruments. If we had sufficient power, with tight confidence intervals for all estimates, the test would tell us something about the ‘true’ effect. In our case, with low power, we argue the focus should be more on the relative magnitude of the estimates and on whether the (OLS, FE and IV) assumptions for causal inference are likely to be met.

We show that, in the context of this research question, the use of the two non-genetic instrument sets is likely to violate the IV assumptions due to their association with many child and family background characteristics that are also associated with the outcome of interest. This therefore suggests that maternal BMI and children's lagged adiposity should not be used as instrumental variables, as they are likely not to meet the exclusion restrictions required of a valid instrument. As discussed above however, this argument does not necessarily generalise to all other research questions examining the effects of fat mass, but more specifically relates to this context, where the main concern relates to unobserved confounding, rather than reverse causation.

## 6 Conclusions

The literature that examines the relationship between child adiposity (or BMI) and educational outcomes generally finds mixed results. It is possible that the differences between studies are overstated: there may be no true difference in associations across studies, but *p*-values for associations may vary due to different sample sizes. Differences between studies may also arise because of variations in associations in different populations, the use of different conditioning variables, or different methodologies. In addition to an OLS, studies generally either specify an individual FE approach and/or an IV specification. Using one common dataset, this paper compares the different approaches, discusses their appropriateness and contrasts the findings. Within the IV approach, we distinguish between three sets of instruments for child adiposity, all of which have been applied in this literature: mother's pre-pregnancy BMI and BMI squared, the child's lagged adiposity categories, and the child's genetic markers.

OLS results show that more adipose children perform worse in school tests compared to their leaner counterparts. These findings are robust to an individual FE specification. We show that the IV results differ depending on the instrument set chosen. Accounting for the endogeneity of fat mass using maternal pre-pregnancy BMI yields large negative estimates, suggesting that fat mass decreases school outcomes, possibly by a greater magnitude than that observed using OLS. Using children's lagged fat mass to instrument for current fat mass leads to (patterns of) estimates that are very similar to the OLS findings, but with slightly larger standard errors. Accounting for the endogeneity of adiposity using the genetic markers shows somewhat ambiguous results, with point estimates that are sometimes smaller, sometimes larger than the OLS estimates. With the large standard errors however, we cannot reject the null of no effect.

The different approaches make different assumptions, which may or may not be valid in this context. Despite our large number of control variables, the OLS estimates are likely to be subject to residual confounding. Likewise, the FE approach does not deal with reverse causation, nor does it deal with time-varying unobservables that affect both fat mass and child outcomes. Examining the two non-genetic instrument sets, we show that they are associated with several child and family background characteristics that are also associated with children's educational outcomes. This casts doubt on their appropriateness as an IV, suggesting that – in this context, where the main concern relates to unobserved confounding rather than reverse causation – they do not satisfy the exclusion restriction criteria required for a valid instrument. The use of genetic variants as instrumental variables in turn may violate the exclusion restriction through the variants’ unknown mechanisms – possible pleiotropy or linkage disequilibrium – although the evidence suggests that these are unlikely to play a role here. In addition, we show that the genetic variants are generally unrelated to the set of child and family background characteristics that are associated with children's educational attainment. Taken together, this suggests that the use of carefully chosen genetic variants as instrumental variables is least likely to obtain biased causal effects.

Nevertheless, our analyses show that most estimates cannot be statistically distinguished from OLS, nor from each other. Statistically speaking, this might suggest that we should rely on the OLS estimates, rather than the IV. However, we are cautious about this interpretation, as the power of some of our analyses is low, particularly for the analyses that use the genetic variants as instruments. If we had sufficient power, with tight confidence intervals for all estimates, the test would tell us something about the ‘true’ effect. In our case, with low power, we argue the focus should be more on the relative magnitude of the estimates and on whether the (OLS, FE and IV) assumptions for causal inference are met.

Taking account of the different ways in which the above methods are likely to have violated key assumptions when addressing causality, and comparing the point estimates from the different approaches, we conclude that fat mass is unlikely to be causally related to academic achievement in adolescence.

## Appendix A A brief introduction to genetics

Each cell in the human body contains a nucleus in which most DNA is kept. DNA is stored in structures called chromosomes, where each chromosome contains a single continuous piece of DNA. All cells in the human body apart from gametes (i.e. germ cells) contain 46 chromosomes, organised into 23 chromosome pairs: one copy of chromosome 1–22 from each parent, plus an X-chromosome from the mother and either an X or a Y chromosome from the father.

Locations (or loci) where DNA varies between people are called polymorphisms. The most commonly studied form of polymorphism is a Single Nucleotide Polymorphism (SNP): a single base-pair variation in a DNA locus. As chromosomes come in pairs, humans have two base-pairs at each locus, called alleles. These alleles can either be the same or different. The term genotype is used to describe the specific set of alleles inherited at a particular chromosome locus. For example, individuals can have one of three genotypes of the FTO SNP (one of the genetic variants used here): they can be homozygous for the common allele (TT), heterozygous (AT), and homozygous for the rare allele of *FTO* (AA). The visible or measurable effect of a particular genotype is called the phenotype.

## Footnotes

1 BMI is defined as weight in kg divided by height in metres squared. Obesity refers to a BMI above 30.

2 Mendelian randomization refers to the random assignment of an individual's genotype from his/her parental genotypes that occurs at conception. Other studies in the economics literature that have used genetic markers as instrumental variables for child or adolescent BMI include Ding et al. (2006), Fletcher and Lehrer (2008), and Norton and Han (2008). Their instruments are a set of variants generally associated with neurotransmitters. As these are unlikely to satisfy the conditions that need to be met for genetic markers to be used as instruments, we do not discuss them further. A recent symposium in *Health Economics* however, specifically examines the use of neurotransmitter-related variants (Cawley et al., 2011) and other genetic variants (von Hinke Kessler Scholder et al., 2011b) in IV specifications, discussing the use of genetic variation both across the population and within families (Fletcher, 2011).

3 See www.bristol.ac.uk/alspac for more a detailed description of the representativeness of the sample, its enrolment, and response rates.

4 The IV specification that uses genetic variants requires us to drop non-white children, as allele frequencies are known to vary across ethnic groups. Any systematic relationship between the allele frequency and the outcome of interest in these different sub-populations can lead to an association between the two at the population level without an actual causal relationship, violating the IV assumptions. This situation is also known as population stratification. To make the analyses comparable across the different approaches, we drop all non-white children. See Appendix A for definitions of some of the terms used in genetics.

5 93 percent of English children attend state schools.

6 See for example Frayling et al., 2007, Hinney et al., 2007, Loos et al., 2008, Willer et al., 2008, Hunt et al., 2008, Thorleifsson et al., 2009, Meyre et al., 2009, Heard-Costa et al., 2009. The rs-number is an identification tag that uniquely positions the polymorphism in the genome.

7 For a brief overview of the genetics terms used here, see Appendix A.

8 Note that, in the absence of population stratification (see footnote 4), controlling for covariates is not required in Mendelian randomization studies, and may lead to biased estimates. The reason is that, as the variants are randomly assigned *at conception*, any characteristic is, in principle, a post-randomization variable, and – with that – may be affected by the instrument. If the instrumented risk factor has multiple causal effects, or if the outcome of interest has a causal effect of its own, adjusting for such post-treatment variables may lead to biased estimates of the causal effect of interest (von Hinke Kessler Scholder et al., 2011a).

9 Under the independence assumption, and in a situation where the instrumented risk factor and outcome do not (directly or indirectly) affect the covariates, the unadjusted and adjusted IV estimates should be similar.

10 Family income is an average of two observations (when the child is aged 3 and 4) and is in 1995 prices. It is equivalised (i.e. adjusted for family size and household composition) using the OECD equivalence scale to allow for a comparison of incomes for all households. The educational indicators are: less than ordinary (O) level, O-level only, advanced (A) level that permits higher educational study, and having a university degree. We use the standard UK classification of social class based on occupation (professional (I), managerial/technical (II), non-manual skilled (IIInm), manual skilled (IIIm), semi-skilled (IV) and unskilled (V)). IMD is based on six deprivation domains, including health deprivation and disability; employment; income; education, skills and training; housing; and geographical barriers to services. Increasing IMD scores indicate greater deprivation. The IMD measure relates to areas containing around 8000 persons.

11 Maternal mental health is measured by the Edinburgh Post-natal Depression Score (EPDS) and Crown-Crisp Experimental Index (CCEI) at 18 weeks gestation. EPDS indicates to what extent the mother is at risk of perinatal depression; CCEI captures a broader definition of mental health, measuring general anxiety, depression and somaticism. Higher scores mean the mother is more affected. The mother's ‘teaching score’ is constructed from questions that measure whether the mother is involved in teaching her child (depending on the child's age) songs, the alphabet, being polite, etc. We use an average score from three measures at ages 18, 30 and 42 months to capture longer-term involvement. Likewise, a variable is included indicating whether the mother reads/sings to the child, allows the child to build towers/other creations, etc., measured at age 24 months. Finally, we also account for the extent to which parents engage in active (outdoor) activities with their children, such as going to the park or playground and going swimming.

12 Although the differences between the original and estimation sample suggest that sample selection may be important, it is likely to affect our different specifications in the same way. Hence, it does not affect the validity of our comparison study.

13 The contextual variables include: child birth weight, age in months, number of older and younger siblings under 18, log family income and its square, mother's and mother's parents’ educational level, whether the child was raised by the natural father, family social class, maternal age at birth, parents’ employment status, and IMD. Indicators for parental health and behaviour include: mother's smoking and drinking during pregnancy, breastfeeding, mother's ‘locus of control’, two measures of maternal mental health (EPDS and CCEI), parental involvement or interest in the child's development, and parents’ engagement in active (outdoor) activities with their child.

14 We combine the independence and exclusion assumptions here, though they are discussed separately in Angrist et al. (2000). For a more detailed discussion of independence as distinct from exclusion within a Mendelian randomization framework, as well as the assumptions of monotonicity, a non-zero effect of the instrument on treatment, and the stable unit treatment value assumption (SUTVA), see von Hinke Kessler Scholder et al. (2011a).

15 Studies that examine the effects of body size on adult labour market outcomes have also used sibling's BMI as IV (e.g. Cawley, 2004, Norton and Han, 2008). We do not observe siblings in the data used here. Other studies have used offspring BMI as an instrument for parental BMI (Cawley, 2000, Davey Smith et al., 2009, Cawley and Meyerhoefer, 2012). Too few of our cohort members have children of their own to examine this in more detail.

16 We use the term ‘reverse causality’ loosely here, as current outcomes cannot have affected previous adiposity. A more precise description would be that there is confounding by (in this example) earlier education (see also Flegal et al., 2011).

17 Our data shows a correlation between 0.935 and 0.960 for BMI measured one year apart. This falls to 0.718 for BMI measured eight years apart, or 0.714 for fat mass measured seven years apart.

18 von Hinke Kessler Scholder et al. (2011a) include a more detailed discussion of the specific conditions that need to be examined in Mendelian randomization experiments and elaborate on the validity of these variants in particular. We do not discuss these here, as this is beyond the scope of this paper: it is not our aim to provide an examination of the conditions for the correct use of genetic markers. The main focus of the current article is to compare the different identification strategies adopted in this literature and to contrast their findings.

19 As discussed in Cawley et al. (2011) and von Hinke Kessler Scholder et al. (2011b) for example, genes related to neurotransmitter-related variants are implicated in many neurological processes, making it difficult to argue that they can be used as valid instruments for one specific risk factor without being associated with others that could plausibly influence the outcome of interest. The genetic variants *FTO* and *MC4R* are therefore arguably better than the neurotransmitters used in the previous literature.

20 With data on a large number of fat mass-related variants, we will be able to examine the possibility of pleiotropy and LD with increased certainty. More specifically, we can specify multiple IV models, each using different independent combinations of these variants. If each of these sets predict the same causal effect, this is very unlikely to be due to some common pleiotropy or LD across the different sets of variants, assuming that the different variants are located on different chromosomes and affect the trait via different pathways (Davey Smith, 2011, Palmer et al., 2012). A large number of variants are currently known to affect fat mass and BMI. Including these in future analyses will greatly improve its power, leading to much smaller standard errors.

21 Using a two-sided binomial probability test at the 10% level, a comparison of the *observed* versus *expected* number of significant correlations suggests that the non-genetic instruments show a greater association with covariates than that expected by chance (*p* < 0.000001 both for maternal BMI and child's lagged BMI). For *FTO* and *MC4R*, the p-values equal 0.73 and 0.03 respectively, suggesting that *MC4R* also shows a slightly higher correlation than that expected by chance; we do not have any prior suppositions as to why this may be the case.

22 Sabia (2007) uses mother's and father's self-reported obesity status. However, as father's height and weight is only known for a much smaller sample, we take Averett and Stifel's (2010) approach and use mother's BMI and its square. Kaestner and Grossman (2009) regress the *change* in educational attainment on the level of child BMI, using the child's 4-year lagged BMI categories as the instruments. To make the analysis more comparable across our alternative instrument sets, we use the *level* of educational performance as the dependent variable.

23 Due to a smaller sample size, the estimates are slightly different from a previous paper that uses the ALSPAC data and Mendelian randomization methods (von Hinke Kessler Scholder et al., 2011a); however, the confidence intervals overlap.

24 The inclusion of school fixed effects leads to similar findings as those described above. Using (standardised) BMI rather than fat mass also gives similar estimates to those presented.

## References

[bib0005] Angrist J., Graddy K., Imbens G. (2000). The interpretation of instrumental variables estimators in simultaneous equation models with an application to the demand for fish. Review of Economic Studies.

[bib0010] Averett S.L., Stifel D.C. (2010). Race and gender differences in the cognitive effects of childhood overweight. Applied Economics Letters.

[bib0015] Burkhauser R.V., Cawley J. (2008). Beyond BMI: the value of more accurate measures of fatness and obesity in social science research. Journal of Health Economics.

[bib0020] Burkhauser R.V., Cawley J., Schmeiser M. (2009). The timing of the ruse in U.S. obesity varies with the measure of fatness. Economics and Human Biology.

[bib0025] Case A., Paxson C. (2008). Stature and status: height, ability and labor market outcomes. Journal of Political Economy.

[bib0030] Cawley J. (2000). An instrumental variables approach to measuring the effect of body weight on employment disability. Health Services Research.

[bib0035] Cawley J. (2004). The impact of obesity on wages. Journal of Human Resources.

[bib0040] Cawley J., Han E., Norton E. (2011). The validity of genes related to neurotransmitters as instrumental variables. Health Economics.

[bib0045] Cawley J., Meyerhoefer C. (2012). The medical care costs of obesity: an instrumental variables approach. Journal of Health Economics.

[bib0240] Cawley J., Spiess K. (2008). Obesity and skill attainment in early childhood. Economics and Human Biology.

[bib0050] Church C., Moir L., McMurray F., Girard C., Banks G., Teboul L., Wells S., Brüning J., Nolan P., Ashcroft F., Cox R. (2010). Overexpression of FTO leads to increased food intake and results in obesity. Nature Genetics.

[bib0055] Davey Smith G., Ebrahim S. (2003). ‘Mendelian randomization’: can genetic epidemiology contribute to understanding environmental determinants of disease?. International Journal of Epidemiology.

[bib0060] Davey Smith G., Sterne J.A.C., Fraser A., Tynelius P., Lawlor D.A., Rasmussen F. (2009). The association between BMI and mortality using offspring BMI as an indicator of own BMI: large intergenerational mortality study. British Medical Journal.

[bib0065] Davey Smith G. (2011). Use of genetic markers and gene-diet interactions for interrogating population-level causal influences of diet on health. Genes and Nutrition.

[bib0070] Ding W., Lehrer S.F., Rosenquist J.N., Audrain-McGovern J. (2006). The impact of poor health on education: new evidence using genetic markers. Journal of Health Economics.

[bib0075] Flegal K., Graubard B., Williamson D., Cooper R. (2011). Reverse causation and illness-related weight loss in observational studies of body weight and mortality. American Journal of Epidemiology.

[bib0080] Fletcher, J.M., Lehrer., S.F., 2008. Using Genetic Lotteries within Families to Examine the Causal Impact of Poor Health on Academic Achievement. NBER Working paper 15148.

[bib0085] Fletcher J. (2011). The promise and pitfalls of combining genetic and economic research. Health Economics.

[bib0090] Frayling T.M., Timpson N.J., Weedon M.N. (2007). A common variant in the FTO gene is associated with body mass index and predisposes to childhood and adult obesity. Science.

[bib0095] Freathy R.M. (2008). Common variation in the FTO gene alters diabetes-related metabolic traits to extent expected, given its effect on BMI. Diabetes.

[bib0100] Golding J., Pembrey J., Jones M.R., ALSPAC Study Team (2001). ALSPAC—the Avon longitudinal study of parents and children. I. Study methodology. Paediatric and Perinatal Epidemiology.

[bib0105] Hamermesh D.S., Biddle J.E. (1994). Beauty and the labor market. American Economic Review.

[bib0110] Heard-Costa N., Zillikens M.C., Monda K.L. (2009). NRXN3 is a novel locus for waist circumference: a genome-wide association study from the CHARGE Consortium. PLoS Genetics.

[bib0210] von Hinke Kessler Scholder, S., Davey Smith, G., Lawlor, D.A., Propper, C., Windmeijer, F., 2010. Child Height, Health and Human Capital: Evidence using Genetic Markers. CMPO Working Paper 10/245. 10.1016/j.euroecorev.2012.09.009 PMC4318168 25673883

[bib0215] von Hinke Kessler Scholder, S., Davey Smith, G., Lawlor, D.A., Propper, C., Windmeijer, F., 2011a. Genetic Markers as Instrumental Variables. CMPO Working Paper 11/274.

[bib0220] von Hinke Kessler Scholder S., Davey Smith G., Lawlor D.A., Propper C., Windmeijer F. (2011). Mendelian randomization: the use of genes in instrumental variable analyses. Health Economics.

[bib0115] Hinney A., Nguyen T.T., Scherag A. (2007). Genome wide association (GWA) study for early onset extreme obesity supports the role of fat mass and obesity associated gene (FTO) variants. PLoS One.

[bib0120] Hunt S., Stone S., Xin Y. (2008). Association of the FTO gene with BMI. Obesity.

[bib0125] Kaestner R., Grossman M. (2009). Effects of weight on children's educational achievement. Economics of Education Review.

[bib0130] Lawlor D.A., Clark H., Davey Smith G., Leon D.A. (2006). Childhood intelligence, educational attainment and adult body mass index: findings from a prospective cohort and within sibling-pairs analysis. International Journal of Obesity.

[bib0135] Lawlor D.A. (2010). The association of general and central adiposity, and change in these in childhood with cardiovascular risk factors in adolescence: a prospective cohort study. British Medical Journal.

[bib0140] Lewis S. (2010). Associations between an obesity related genetic variant (FTO rs9939609) and prostate cancer risk. PLoS One.

[bib0145] Li X. (1995). A study of intelligence and personality in children with simple obesity. International Journal of Obesity and Related Metabolic Disorders.

[bib0150] Loos R.J., Lindgren J.M., Li S. (2008). Common variants near MC4R are associated with fat mass, weight and risk of obesity. Nature Genetics.

[bib0155] Meyre D., Delplanque J., Chevre J-C. (2009). Genome-wide association study for early-onset and morbid adult obesity identifies three new risk loci in European populations. Nature Genetics.

[bib0160] Nordestgaard B.G. (2010). Increased body mass index and increased risks of ischemic heart disease: using genome wide association results to estimate causal effects with Mendelian randomization. Circulation.

[bib0165] Norton E.C., Han E. (2008). Genetic information, obesity and labor market outcomes. Health Economics.

[bib0170] OECD (2007). Health at a Glance 2007: OECD Indicators.

[bib0175] Palmer T.M., Lawlor D.A., Harbord R.M., Sheehan N.A., Tobias J.H., Timpson N.J., Davey Smith G., Sterne J.A. (2012). Using multiple genetic variants as instrumental variables for modifiable risk factors. Statistical Methods in Medical Research.

[bib0180] Sabia J.J. (2007). The effect of body weight on adolescent academic performance. Southern Economic Journal.

[bib0185] Sorensen T.I., Sonne-Holm S., Christensen U. (1983). Cognitive deficiency in obesity independent of social origin. Lancet.

[bib0190] Thorleifsson G., Walters G.B., Gudbjartsson D.F. (2009). Genome-wide association yields new sequence variants at seven loci that associate with measures of obesity. Nature Genetics.

[bib0195] Timpson N., Emmet P.M., Frayling T.M. (2008). The fat mass- and obesity-associated locus and dietary intake in children. American Journal of Clinical Nutrition.

[bib0200] Timpson N. (2009). Does greater adiposity increase blood pressure and hypertension risk? Mendelian randomization using the FTO/MC4R genotype. Hypertension.

[bib0205] Timpson N. (2009). How does body fat influence bone mass in childhood? A Mendelian randomization approach. Journal of Bone and Mineral Research.

[bib0225] Wardle J., Carnell S., Haworth C.M.A. (2008). Obesity Associated genetic variation in FTO is associated with Diminished satiety. The Journal of Clinical Endocrinology and Metabolism.

[bib0230] Willer C., Speliotes E.K., Loos R.J. (2008). Six new loci associated with body mass index highlight a neuronal influence on body weight regulation. Nature Genetics.

[bib0235] Zimmerman E. (2009). Fatness-associated FTO gene variant increases mortality independent of fat mass—in cohorts of Danish men. PLoS One.

